# Development of a sensitive microplate assay for characterizing RNA methyltransferase activity: Implications for epitranscriptomics and drug development

**DOI:** 10.1016/j.jbc.2023.105257

**Published:** 2023-09-14

**Authors:** Isaiah K. Mensah, Allison B. Norvil, Ming He, Emma Lendy, Nicole Hjortland, Hern Tan, Richard T. Pomerantz, Andrew Mesecar, Humaira Gowher

**Affiliations:** 1 Department of Biochemistry, Purdue University Center for Cancer Research, Purdue University, West Lafayette, Indiana, USA; 2 Department Biochemistry and Molecular Biology, Thomas Jefferson University, Sidney Kimmel Cancer Center, Philadelphia, Pennsylvania, USA

**Keywords:** NS5, RNA methylation, biotin–avidin, methylation activity, RdRP, Zika virus

## Abstract

RNA methylation is a ubiquitous post-transcriptional modification found in diverse RNA classes and is a critical regulator of gene expression. In this study, we used Zika virus RNA methyltransferase (MTase) to develop a highly sensitive microplate assay that uses a biotinylated RNA substrate and radiolabeled AdoMet coenzyme. The assay is fast, highly reproducible, exhibits linear progress-curve kinetics under multiple turnover conditions, has high sensitivity in competitive inhibition assays, and significantly lower background levels compared with the currently used method. Using our newly developed microplate assay, we observed no significant difference in the catalytic constants of the full-length nonstructural protein 5 enzyme and the truncated MTase domain. These data suggest that, unlike the Zika virus RNA-dependent RNA polymerase activity, the MTase activity is unaffected by RNA-dependent RNA polymerase–MTase interdomain interaction. Given its quantitative nature and accuracy, this method can be used to characterize various RNA MTases, and, therefore, significantly contribute to the field of epitranscriptomics and drug development against infectious diseases.

RNA methylation is one of over 150 RNA modifications identified, leading to an emergence of a field of study called epitranscriptomics (1, 2, 3). The MODOMICS database lists 72 different methyl-group modifications in RNA (4, 5). RNA methylation regulates the post-transcriptional processing of the nascent transcript, which includes RNA splicing, stability, nuclear export, and translation (6, 7, 8, 9, 10, 11, 12, 13, 14), affecting diverse cellular processes, such as DNA repair, miRNA biogenesis, and cellular differentiation (15, 16, 17). The majority of RNA methylations occur on noncoding RNA molecules, such as tRNA, rRNA, and small nuclear RNA (18). These RNA modifications, including C5-methylcytidine, N6-methyladenosine, and N1-methyladenosine, primarily occur on the nucleoside base rather than the sugar–phosphate backbone of the RNA molecule. On the other hand, 2′-O methylation (Nm) is the most common modification in viral RNA and is associated with RNA structure, stability, and interactions (19). These processes are critical for viral replication and antiviral immune responses (20).

The recent development of transcriptome-wide approaches to capture various RNA modifications has improved our understanding of the role of RNA methylation in fundamental cellular processes essential for normal development (1). Nevertheless, compared with other enzymes that catalyze group transfer reactions, such as DNA methyltransferases and kinases (21), the enzymatic properties and the molecular mechanism of RNA MTases are relatively less understood (22). This could partly be the drawback of the tedious and inaccurate nature of the current *in vitro* methods used to characterize RNA MTase enzymes. In this study, we used Zika virus (ZIKV) nonstructural protein 5 (NS5) RNA MTase to develop a robust medium throughput microplate assay for enzymatic characterization of RNA MTases.

ZIKV is a member of the Flaviviridae family. The viral genome replication is driven by an NS5, containing two active domains (23). The polymerase domain of NS5 makes double-stranded RNA from the single-stranded RNA genome, followed by transcription and replication. The MTase domain of NS5 uses AdoMet as a methyl group donor to methylate the guanosine cap (GpppN) and internal adenosine residues of the transcript. Studies have revealed that N7 methylation of the cap is necessary for RNA stability and recognition by the viral elongation factors, and its inhibition leads to severe replication defects in the host. Internal adenosine methylation occurs at the 2′-O position on the ribose to protect the viral genome against host recognition. Therefore, inhibiting Nm attenuates viral propagation in the host (24, 25, 26, 27, 28).

Identifying inhibitors for the MTase domain of flavivirus family members is an important step in developing potential antiviral therapies. *In vitro* studies using RNA methylation assays and recombinant MTase enzymes have shown that inhibitors of AdoMet binding, such as Sinefungin and AdoHcy, can suppress NS5 activity (25, 26, 29, 30, 31). These assays utilize radioactive [methyl-^3^H]-AdoMet, ideal for detecting methyl group incorporation. The reaction mixture containing radioactively labeled methylated RNA is spotted onto diethylaminoethyl (DEAE) cellulose paper strips, which are washed with buffer and ethanol. The filter strips are dried and soaked in scintillation fluid. The total incorporated radioactivity is then measured in a liquid scintillation counter (32). Although the DEAE paper strongly binds nucleic acids (RNA and DNA), it nonspecifically binds a significant amount of free [methyl-3H]-AdoMet. This results in a high radioactivity background, which obscures the measurement of low methylation activity (33). In addition, DEAE cellulose requires each sample to be processed individually, limiting the processing power of the assay.

Here, we demonstrate a convenient and reproducible method for measuring RNA MTase activity. The assay utilizes a 3′-biotinylated (Bt) RNA substrate that can be immobilized on avidin-coated 96-well plates. In an *in vitro* methylation reaction, we used the ZIKV NS5–MTase enzyme to transfer the radiolabeled-CH_3_ group from ^3^[H]-AdoMeT to an internal adenine of the Bt-RNA substrate. The reaction mix is transferred into avidin-coated wells of an ELISA plate that provide the binding surface to the Bt-RNA. The unbound reactants and coproducts are washed out, and methyl groups transferred to the bound RNA substrate are quantified directly on the ELISA plate by liquid scintillation counting using a MicroBeta^2^ plate reader. To demonstrate the feasibility of our assay, we determined the kinetic properties of the full-length or truncated ZIKV MTase and showed the inhibition kinetics of AdoHcy. Previous studies have shown that the MTase domain affects the RdRP activity in ZIKV NS5 enzymes (34, 35, 36). The MTase domain interacts and stabilizes motif F in the RdRp domain necessary for the catalytic activity of the RdRp domain (29, 37, 38, 39, 40). Our assay showed no significant difference in the catalytic constants of the full-length and truncated NS5–MTase domain suggesting the MTase activity is not influenced by the MTase–RdRP interdomain interaction. Our improved method can be used to screen small-molecule inhibitors and determine their inhibition constants, for example, IC_50_ and *K*_i_ values, that are extremely valuable for iterative structure-based design studies and, ultimately, drug development.

## Results

### Enzymatic activity of truncated ZIKV MTase domain using filter-binding assays

**Figure 1:**
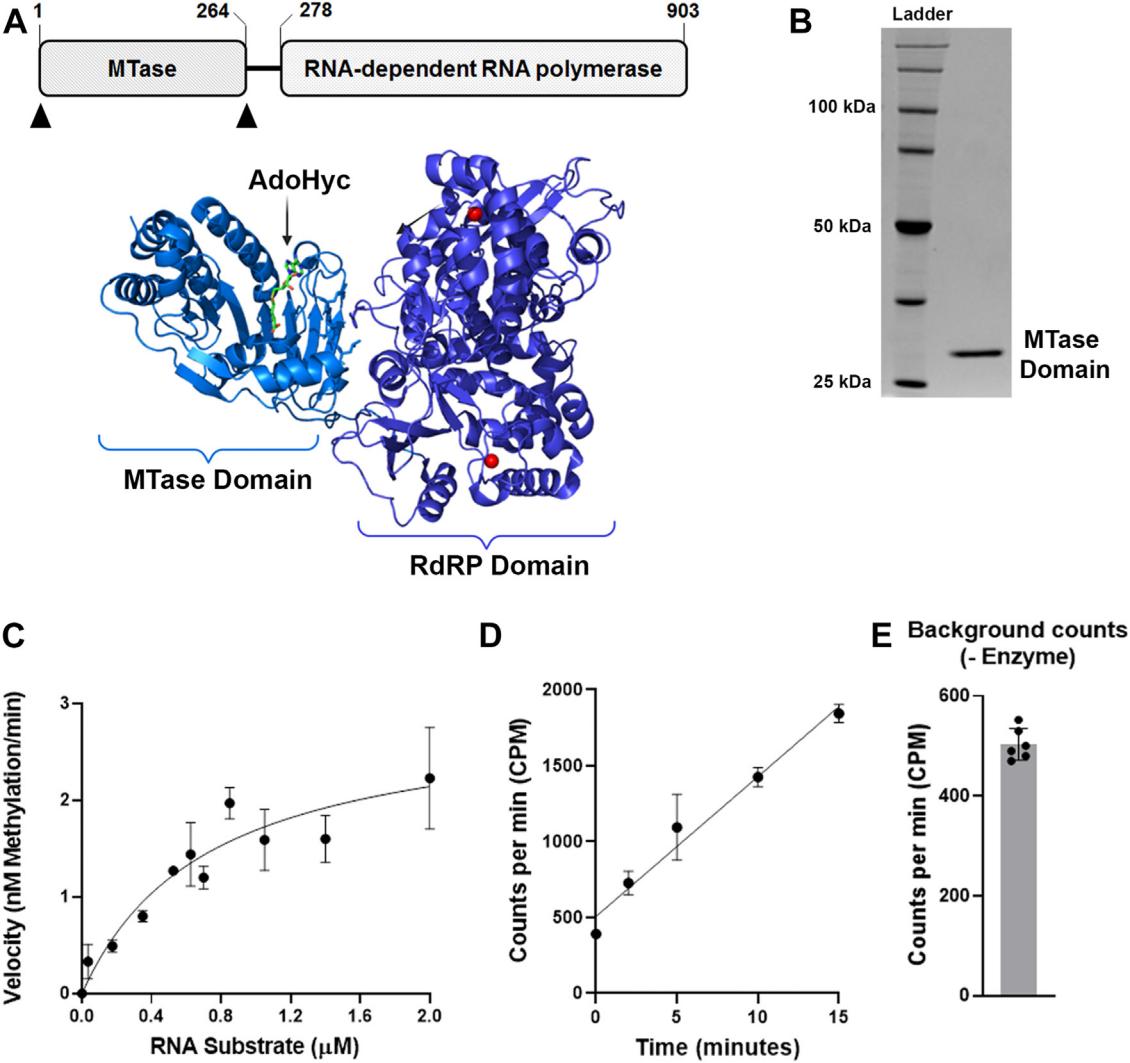
**Characterization of the truncated ZIKV NS5–MTase domain using the DEAE filter assay.***A*, schematic showing the NS5 enzyme of ZIKV containing an N-terminal MTase domain fused to a C-terminal RdRp domain through a linker. Crystal structure of ZIKV NS5 from PDB: 5TFR showing the MTase domain and the RdRP domain. *B*, Coomassie-stained SDS-PAGE gel of the purified His-tagged recombinant ZIKV MTase domain. The isolated MTase domain (1–264) was used for biochemical characterization. *C*, steady-state kinetic analysis of the MTase activity. Initial velocities of methylation reactions performed at substrate concentrations ranging from 35 nM to 2 μM were plotted as the concentration of methyl groups incorporated. The data were fit to the Michaelis–Menten equation to obtain the catalytic constants. *V*_max_ was calculated as 2.92 ± 0.12 nM/min, and *K*_*M*_ was determined as 0.73 ± 0.24 μM. *D*, MTase activity assay with 5 μM enzyme and 0.35 μM of RNA substrate showed high CPM at zero time point. *E*, the background level of [^3^H] nonspecifically bound to the DEAE filter was detected by performing an MTase assay with [methyl-^3^H]-AdoMet and Bt-RNA substrate without the enzyme. For (*C*), the data points and errors bars shown are the average ± SEM (n ≥ 3), where n is slope from one reaction kinetics. For (*D*) and (*E*), the data points and error bars are the average ± SD (n ≥ 3). Bt, biotinylated; DEAE, diethylaminoethyl; MTase, methyltransferase; NS5, nonstructural protein 5; RdRp, RNA-dependent RNA polymerase; ZIKV, Zika virus.

The Zika viral genome encodes for NS5 that contains an N-terminal RNA methyltransferase domain and a C-terminal RNA-dependent RNA polymerase (RdRp) domain (Fig. 1*A*). The recombinant truncated domain (NS5–MTase) methyltransferase domain is a ∼30 kDa protein known to be active in the absence of the RdRp domain (40). We purified the His-tagged recombinant NS5–MTase (Fig. 1*B*) and validated its enzymatic activity. Briefly, methylation reactions were carried out using single-stranded RNA containing 27 adenosine residues as a substrate and [methyl-^3^H]-AdoMet as methyl donor coenzyme. The conventional DEAE filter binding was used to perform kinetic assays at varying RNA concentrations from 0.035 to 2.0 μM (Fig. S1). Michaelis–Menten constants were derived from the reaction rates using GraphPad Prism (GraphPad Software, Inc). The *K*_*M*_ and turnover rate constant (*K*_cat_) values were (7.3 ± 2.4) × 10^−7^ M and (10.0 ± 0.5) × 10^−6^ S^−1^, respectively. The catalytic efficiency was expressed as *K*_cat_/*K*_*M*_ ∼14 M^−1^ S^−1^ (Fig. 1*C*). This *K*_*M*_ value is similar to what has been reported in previous studies (26), demonstrating the recombinant enzyme's high quality and specific activity.

The commonly used DEAE filter-binding assay, however, has several disadvantages. For example, the kinetics experiments show high CPM values at the zero time point, suggesting either burst kinetics or a high background level (Fig. 1*D*). However, the control experiment with [methyl-^3^H] AdoMet and Bt-RNA in a reaction mix without enzyme confirmed the high background of ∼500 CPM (∼7 nM, methyl groups), potentially because of the nonspecific binding of AdoMet to the DEAE filter paper (Fig. 1*E*). Moreover, each reaction is individually processed on small paper strips, which tend to tear off during washes making the assay cumbersome and inaccurate.

### The biotin–avidin microplate assay to detect RNA methyltransferase activity

**Figure 2:**
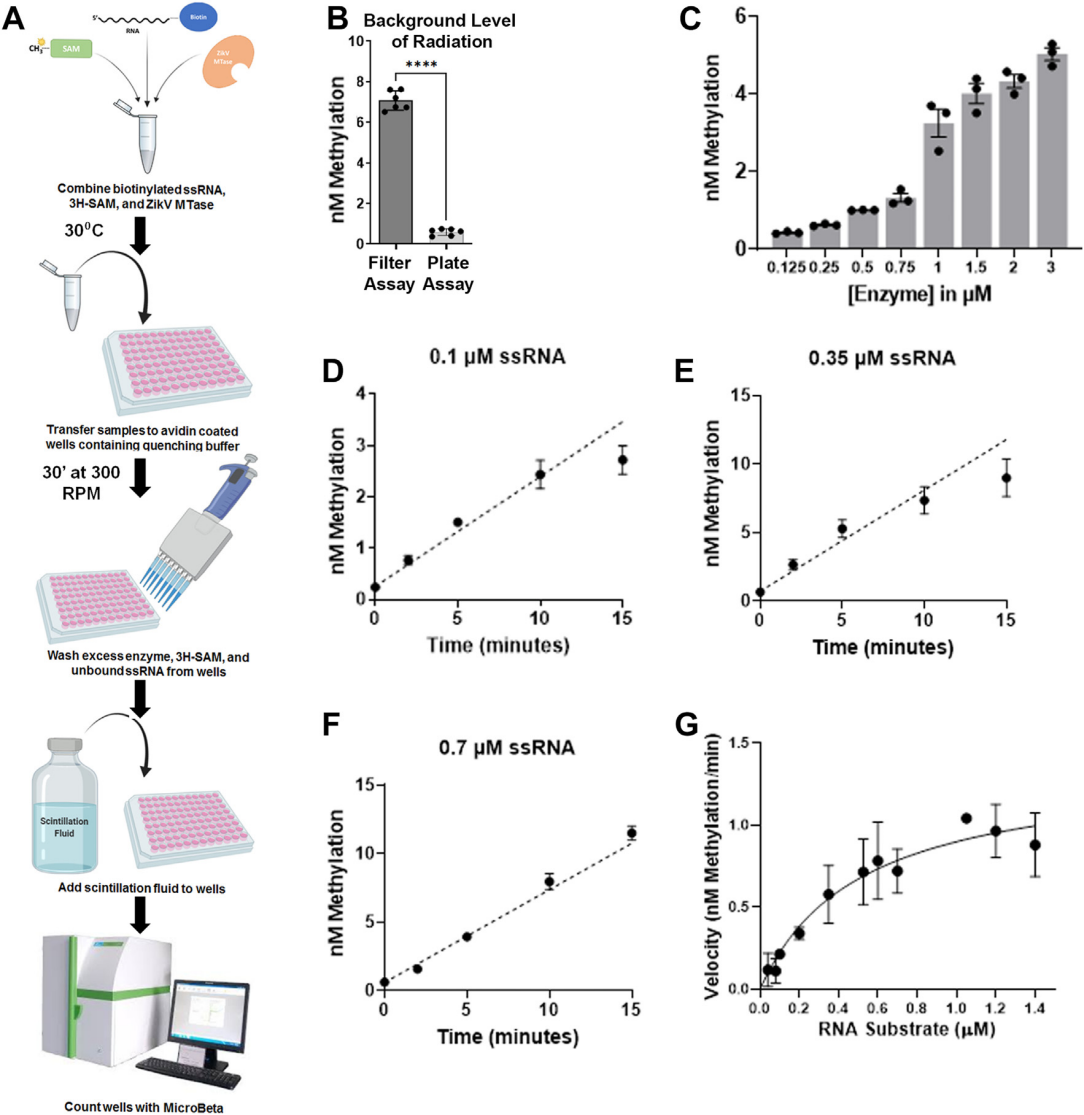
**Characterization of the truncated ZIKV NS5–MTase domain using the microplate assay.***A*, schematic representation of the steps involved in the biotin–avidin microplate assay. *B*, background level of [^3^H] was detected by performing an MTase assay with [methyl-^3^H]-AdoMet and Bt-RNA substrate without the enzyme. *C*, assays were performed using 1 μM Bt-RNA and 0.7 μM [methyl-^3^H]-AdoMet with varying enzyme concentrations. *D*–*F*, representative primary plots of MTase reaction kinetics at 0.1, 0.35, or 0.7 μM RNA substrate and 5 μM enzyme. The reaction was stopped and measured at 2, 5, 10, and 15 min. The initial rate of reaction was determined using linear regression. *G*, initial velocities from methylation kinetics (Nm methylation/min) were plotted against their respective RNA concentration. The data were fit to the Michaelis–Menten equation to determine kinetic constants. *V*_max_ was calculated as 1.36 ± 0.28 nM/min, and *K*_*M*_ was determined as 0.51 ± 0.08 μM. For (*B*–*F*), the data points and error bars are the average ± SD (n ≥ 2). For (*B*), *p* values were derived from Student's *t* test: ∗∗∗∗*p* < 0.0001. The data shown for (*G*) are the average ± SEM (n ≥ 3), where n is slope from one reaction kinetics. Bt, biotinylated; MTase, methyltransferase; Nm, 2′-O-methylation; NS5, nonstructural protein 5; ZIKV, Zika virus.

We developed a microplate RNA MTase activity assay to circumvent the high background and low processing power of the DEAE filter binding method. A similar method was previously developed for measuring DNA and histone methylation activity (33, 41). The method uses avidin-coated 96-well microtiter ELISA plates and Bt-tagged RNA substrate (Fig. 2*A*). In a methylation reaction, ZIKV NS5–MTase transferred the radiolabeled -CH3 group from [methyl-^3^H] AdoMet to the Bt RNA substrate. The methylation reaction was quenched in the avidin-coated ELISA plate in a quenching buffer containing high salt and unlabeled AdoMet. The Bt-RNA was immobilized on the plate through the biotin–avidin interaction. Next, the unbound reaction components, including unincorporated [methyl-^3^H]-AdoMet, were washed using the high salt buffer. Finally, scintillation fluid was added to the wells, and radioactivity incorporated with the -CH_3_ group on the immobilized RNA was measured using a MicroBeta^2^ plate reader by scintillation counting.

Compared with the DEAE filter assay, the biotin–avidin microplate assay has 8 to 10 times less background (<1 nM), increasing the sensitivity of the assay (Fig. 2*B*). In addition, the Z-factor for the microplate assay is 0.66, compared with −0.32 for the filter assay (Table 1) (42). A value between 0.5 and 1.0 indicates that the assay is robust and of high quality, therefore, appropriate for screening purposes (42). Further assay validation was done by confirming the linearity of the signal at varying enzyme concentrations (Fig. 2*C*). To test the fidelity of the method, we determined the catalytic constants of NS5–MTase using the microplate assay. We performed kinetic assays at varying RNA concentrations from 0.04 to 1.4 μM (Fig. S2). Reaction rates were used to derive the Michaelis–Menten constants using GraphPad Prism (Fig. 2, *D*–*F*). *K*_*M*_ and *K*_cat_ values were computed to be (5.1 ± 0.8) × 10^−7^ M and (4.5 ± 0.3) × 10^−6^ S^−1^, respectively. The catalytic efficiency was expressed as *K*_cat_/*K*_*M*_ ∼9 M^−1^ S^−1^ (Fig. 2*G*). The values are in the same range as those previously published (26) and determined by the DEAE filter assay (Fig. 1*C*), confirming the method's reliability. The binding capacity of an avidin-coated well for Bt-RNA was estimated to be around 3 pmol, similar to the previously known binding of Bt-DNA (33). Given that the *K*_*M*_ of most RNA and DNA MTases tends to be in the nanomolar range, the binding capacity of the avidin-coated well is sufficient for these measurements.

**Table 1:** Comparison of the DEAE filter to the biotin–avidin microplate assay

Characteristics	DEAE filter	Microplate assay
Enzyme specific	No	No
Fully commercially available	No	No
*Z*-factor	−0.32	0.66
Background	High (∼500 CPM)	Low (∼50 CPM)
Ease of assay	Low	High
Assay type	End point	End point

### Comparing catalytic constants of truncated NS5–MTases with the full-length NS5

**Figure 3:**
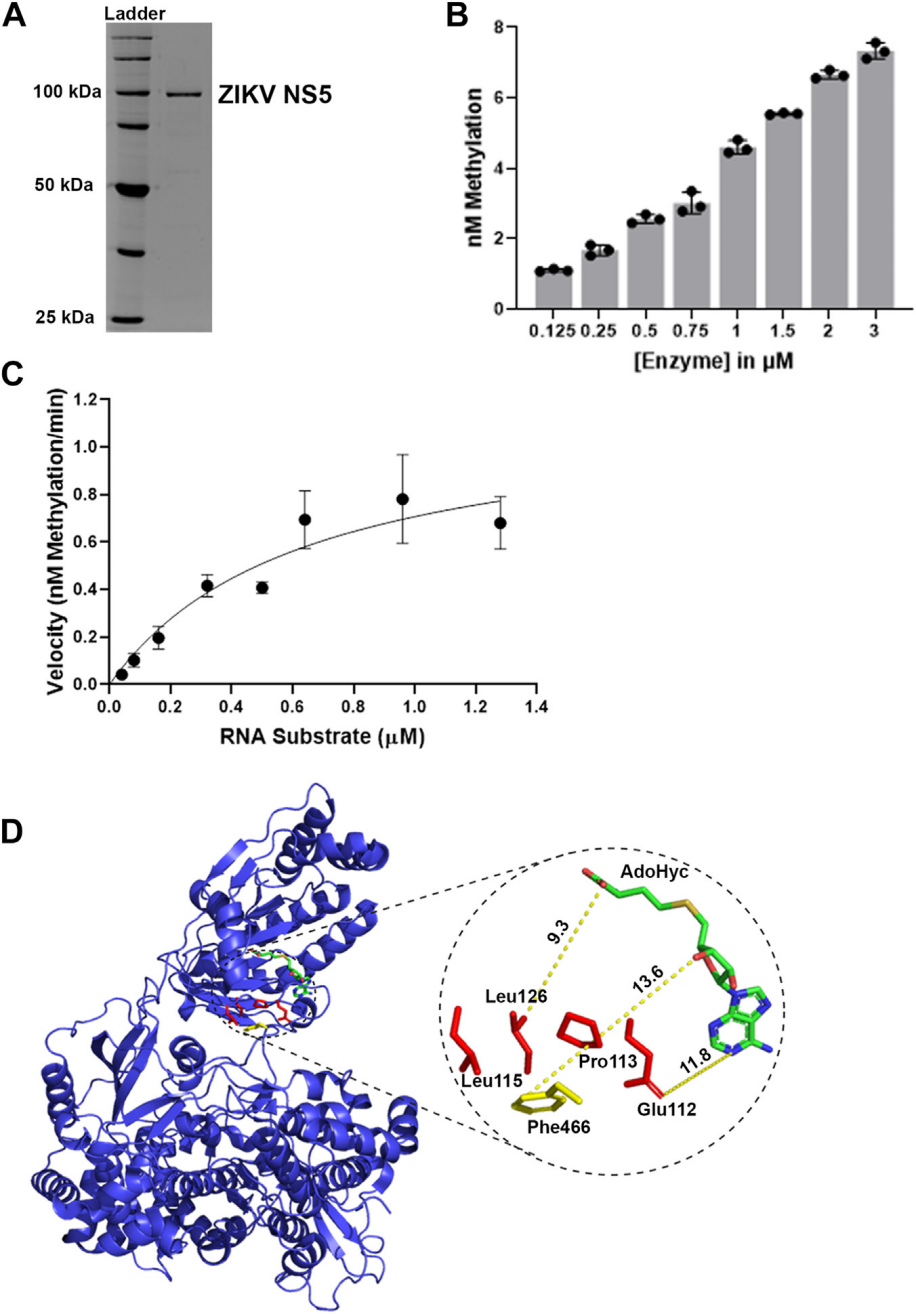
**Characterization of MTase activity of the full-length ZIKV NS5 using the microplate assay.***A*, Coomassie-stained SDS-PAGE gel of the purified His-tagged ZIKV NS5 protein. *B*, RNA methylation activity at varying enzyme concentrations. *C*, for the Michaelis–Menten plot, slopes from methylation kinetics (Nm methylation/min) were plotted against their respective RNA concentration. The data were fit with a nonlinear Michaelis–Menten formula to determine kinetic constants. *V*_max_ was calculated as 1.16 ± 0.25 nM/min, and *K*_*M*_ was determined as 0.64 ± 0.08 μM. The data shown are the average ± SEM (n ≥ 3). *D*, crystal structure of ZIKV NS5 from PDB: 5TFR. The *yellow* and *red sticks* represent interdomain interaction residues. The *green stick* structure is AdoHcy. The zoomed-in view of the F motif highlights the distance between bound AdoHcy and the interaction surface residues. For (*B*), the data points and error bars are the average ± SD (n ≥ 3). The data shown for (*C*) are the average ± SEM (n ≥ 3), where n is slope from one reaction kinetics. MTase, methyltransferase; Nm, 2′-O-methylation; NS5, nonstructural protein 5; PDB, Protein Data Bank; ZIKV, Zika virus.

The NS5 holoenzyme contains the MTase domain fused with the RdRp domain through a linker (Fig. 1*A*) (23). While the RdRp domain is essential for the replication and propagation of the viral genome, the MTase domain is responsible for RNA capping (43, 44). Studies suggest that the ZIKV MTase domain is required for RNA initiation and nucleotide incorporation of the RdRp enzyme while maintaining the stability and activity of the RdRp elongation complex (34, 40). However, whether the RdRp domain of ZIKV NS5 influences the MTase activity is not known. To test this, we determined the catalytic constants of the RNA MTase activity of the full-length NS5 protein and compared them with those obtained for the truncated MTase domain. The enzyme was purified as previously described, and catalytic activity was tested by measuring enzyme activity at various enzyme concentrations (Fig. 3, *A* and *B*) (40). Similar to the truncated NS5–MTase enzyme, we performed kinetic assays using 5 μM enzyme at varying RNA concentrations from 0.04 to 1.4 μM (Fig. S3). The *K*_*M*_ and *K*_cat_ value for the full-length ZIKV NS5 were computed to be (6.4 ± 0.8) × 10^−7^ M and (3.8 ± 0.2) ×10^−6^ S^−1^, respectively (Fig. 3*C*). The catalytic efficiency was expressed as *K*_cat_/*K*_*M*_ ∼6 M^−1^ S^−1^. Since these values are very similar to the catalytic constants for the truncated NS5–MTase domain, the data suggest little or no effect of the interdomain interaction on the MTase activity. We performed kinetic reactions at lower enzyme concentrations to ensure multiple turnover conditions. However, no difference in the reaction rates was measured (Fig. S4, *A* and *B*). These data suggest that under the experimental conditions (40), where MTase and RdRp interdomain interaction affects the RdRP activity, the catalytic activity of the MTase is not affected. A closer look at the crystal structure of NS5, highlighting the residues involved in the interdomain interaction, shows that the AdoMeT binding site is oriented away from the interaction interface (Fig. 3*D*). Moreover, the interface residues are too far to make any direct contact with AdoMeT supporting our experimental results.

### Microplate assays to determine IC_50_ of AdoHcy

**Figure 4:**
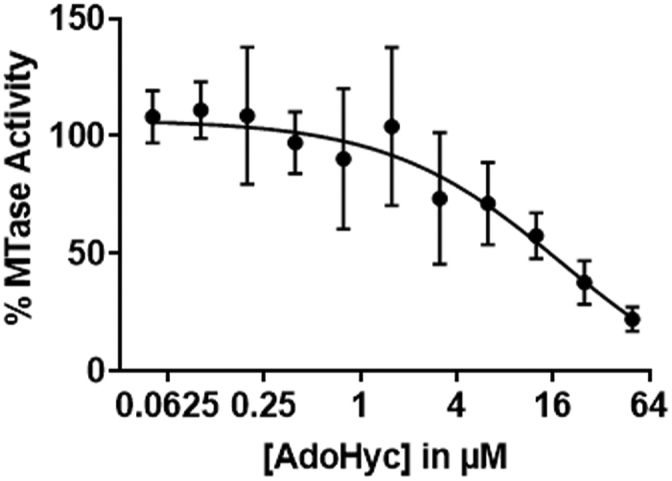
**ZIKV MTase inhibition.** Using biotin–avidin microplate assay, RNA methylation reactions were carried out with varying AdoHcy concentrations in the presence of the A27 RNA substrate. The graph shows the data points, and error bars are the average ±SD from n ≥ 3 independent experiments. MTase, methyltransferase; ZIKV, Zika virus.

Previous biochemical analyses have tested the inhibition of ZIKV NS5–MTase activity by AdoHcy (45). AdoHcy, the byproduct of AdoMet in a methylation reaction, binds efficiently to the AdoMet binding pocket and acts as a competitive inhibitor. To test the sensitivity of the biotin–avidin microplate assay, we monitored ZIKV MTase activity in the presence of varying concentrations of AdoHcy (Fig. 4). MTase activity is inhibited with an IC_50_ of 18 μM, similar to the previously reported value (26). However, because of a low background of the microplate assay compared with conventional DEAE filter assays, small changes in MTase activity were detectable at higher concentrations of AdoHcy. We calculated the $Z^{\prime }$-factor to be 0.59 suggesting it to be a high-quality inhibitor screening assay. Compared with the DEAE filter assay, the microplate assay can screen inhibitors at a medium throughput, thus improving its utility for discovering specific small-molecule inhibitors of RNA MTases.

## Discussion

This work focuses on developing a medium-throughput method to characterize diverse RNA MTases and measure their activity with high accuracy and sensitivity. Compared with the conventional DEAE filter-binding assay, the microplate assay has reduced radioactive [^3^H] background by >90%, thus increasing the sensitivity of our assay.

However, similar to other MTase assays, our assay also uses a radioactive methyl donor molecule, AdoMet, which is labeled with tritium ^3^[H]. Using radioactive AdoMet in the assay provides several advantages. (1) [methyl-^3^H]-AdoMet allows direct detection of methyl group incorporation into RNA substrates without secondary reactions detecting products of the reactions. (2) [methyl-^3^H]-AdoMet is available at a higher specific activity than other radioactive sources like ^14^[C]-labeled AdoMet, increasing the sensitivity of methyl group incorporation. In addition, [methyl-^3^H]-AdoMet has a longer half-life, allowing for a more extended detection window. However, because of the high compliance costs associated with radioactivity, alternative nonradioactive methods, such as fluorescence-based assays or mass spectrometry–based techniques, are also being explored for detecting RNA methylation in a high-throughput and quantitative manner (46, 47). Although these methods require expensive equipment and are more tedious than microplate assay, they offer advantages in terms of safety and the ability to screen more extensive compound libraries to identify potential inhibitors ((48) and references therein). Nonradioactive AdoMet analogs have been used to label DNA with MTase activity. However, the MTase enzymes do not efficiently incorporate some of these analogs, and others are not commercially available (48, 49, 50). Nevertheless, the development of commercially available nonradioactive AdoMet derivatives, which can replace [methyl-^3^H]-AdoMet, will highly increase the utility of our microplate assay.

We used the microplate assay to characterize the allosteric properties of ZIKV NS5–MTase. Previous studies have shown that an interaction between MTase and RdRP domain stabilizes the F-motif in RdRP, which is essential for RdRP activity. Our data suggested that interdomain interaction between MTase and RdRP has little to no effect on the MTase activity of the NS5 enzyme. Given that A27 RNA may not behave like a natural substrate in the *in vitro* assay, we cannot dismiss that the substrate-induced change *in vivo* may affect the interdomain interaction and activity of both domains. However, the microplate assay is not limited by the use of any specific RNA substrate, except that it must be biotinylated. Therefore, the assay can be used to determine the catalytic activity, substrate specificity, and allosteric properties of diverse RNA MTases. This information will not only improve our understanding of the mechanisms that regulate the epitranscriptome but also facilitate the development of small-molecule inhibitors that could be used as antibiotics or anticancer drugs. Moreover, most recent pandemics were caused by (+) ssRNA viruses like ZIKV and coronavirus (51, 52). The inhibition of the viral RNA methyltransferases has a therapeutic potential since they are necessary for viral propagation inside the host cells. We validated the sensitivity of the microplate assay by performing an AdoHcy inhibition titration. The microplate assay reproducibly detected lower MTase activity in the presence of higher AdoHcy concentrations than previously reported (26). If attached to the robotic arm, the microplate assay can also be utilized to assess the efficacy of a smaller pool of potential inhibitors that pass the primary high-throughput screen and more accurately determine the range of inhibitor dosage used in the *in vivo* assays. Moreover, the MicroBeta^2^ plate reader, with a loading capacity of 14 microtiter plates, adds to the throughput potential of this assay.

## Experimental procedures

### ZIKV MTase expression and purification

DNA corresponding to the methyltransferase protein from ZIKV strain *Aedes africanus/SEN/DakAr41524/1984* was codon-optimized for bacterial expression and subcloned into a pET11a plasmid through BioBasic. The protein construct was also designed to contain an N-terminal histidine tag followed by a tobacco etch virus cleavage site (ENLYFQS). DNA was electroporated into BL21(DE3) (Agilent) cells and plated onto an LB agar media supplemented with 50 μg/ml carbenicillin. Colonies were allowed to grow overnight at 37 °C. A single colony was then used to inoculate 150 ml of LB media supplemented with 50 μg/ml carbenicillin, which was grown overnight at 37 °C while shaking at 200 RPM. The next day, 50 ml of the overnight culture was added to 1 l of LB media supplemented with 50 μg/ml carbenicillin. The 1 l culture was allowed to grow at 37 °C, 200 RPM until the absorbance at 600 nm reached 0.6. The culture was then cooled at 4 °C for 30 min. Protein expression was induced with the addition of 0.25 mM IPTG, and protein expression was carried out for 18 h at 25 °C. Cell pellets were harvested by centrifugation and stored at −80 °C until further use.

Cell pellets were thawed on ice and resuspended in 5 ml buffer A (50 mM Tris [pH 7.5], 500 mM NaCl, 5% glycerol, 5 mM β-mercaptoethanol, and 10 mM imidazole), supplemented with 50 μg/ml DNase and 200 μg/ml lysozyme per 1 g of cells. Resuspended cells were sonicated on ice using a Branson sonifier for 12 min at 60% amplitude (pulse on for 5.5 s and pulse off for 9.9 s). The lysate was then centrifuged at 30,000*g* for 30 min at 4 °C to pellet cell debris and unlysed cells. The resulting supernatant was filtered using a 0.45 μm filter before being loaded onto a 5 ml immobilized metal affinity chromatography column (GE Healthcare) bound with Ni^+2^ and equilibrated in buffer A. After the supernatant was loaded onto the column, the column was washed with buffer A until the UV_280_ reading reached baseline. Protein was eluted from the column using a linear gradient from 0% to 100% buffer B (50 mM Tris [pH 7.5], 500 mM NaCl, 5% glycerol, 5 mM β-mercaptoethanol, and 450 mM imidazole) over 100 ml. About 5 ml fractions were collected over the entirety of the gradient. Fractions containing ZIKV MTase, as assessed using SDS-PAGE, were pooled and incubated at a 25:1 ratio with tobacco etch virus protease to remove the histidine tag. Cleavage was allowed to proceed overnight at 4 °C while the protein was simultaneously dialyzed against 2 l of buffer A. The dialyzed and cleaved protein was spun down at 30,000*g* to remove any protein aggregate, and the resulting supernatant was loaded onto a 5 ml Ni column equilibrated in buffer A. The ZIKV MTase protein was collected, and its purity was analyzed using SDS-PAGE. ZIKV MTase protein was then aliquoted, flash-frozen in liquid nitrogen, and stored at −80 °C until further use.

Full-length NS5 enzyme was purified as described (40).

### Biotin–avidin microplate assay

About 96-well ELISA plates were coated with a 10 μg/ml avidin solution (Sigma; catalog no.: A9275-2MG) made in 0.1 M sodium bicarbonate, pH 9.6. About 100 μl of the avidin solution was transferred in each well, and the plate was stored at 4 °C. The wells were washed with 200 μl of 1× PBS with Tween-20 (PBST) at least five times before use to remove any excess avidin solution not bound to the plate.

Methylation assays to determine kinetic parameters of recombinant ZIKV methyltransferase were performed using 0.33 μM [^3^H]-labeled AdoMet (PerkinElmer; catalog no.: NET155V001MC) and 3′-Bt 27-bp polyadenosine ssRNA substrate. Methylation assays were carried out at various concentrations of RNA and enzyme in methylation buffer (40 mM Tris–HCl, pH 8.0, 1 mM DTT, and 2 μM AdoMet). Potential RNA degradation was prevented by supplementing the reaction mix with an RNase inhibitor (ThermoFisher; catalog no.: EO0381). Reactions were carried out for 2, 5, 8, and 15 min at 30 °C, and reaction aliquots were transferred at specified time points into avidin-coated wells supplemented with the quenching buffer (1 mM unlabeled AdoMet [Sigma; catalog no.: A2408-25MG] in 1× PBST, 0.5 M NaCl, and 0.1 mM EDTA). The reaction mix was incubated for 30 min on a microplate vortex mixer (Fisherbrand) at 300 RPM to allow the binding of Bt-RNA. Next, the wells were washed five times with 1× PBST and 0.5 M NaCl using a multichannel pipette to remove unused [methyl-^3^H]-AdoMet and other unbound reaction components. About 200 μl of scintillation fluid was dispensed in the wells, and the incorporated radioactivity was quantified by scintillation counting using a MicroBeta^2^ plate reader (PerkinElmer; catalog no.: 2450-0010).

### Filter-binding assay

Methylation assays using DE81 filter paper were performed using the previously described standard protocol (32). Briefly, the reactions were quenched with 4 mM AdoMet, and 10 μl of the reaction mixture was spotted on 0.5-inch DE81 filters that were washed 3 to 5 times with 0.2 M ammonium bicarbonate 2 to 3 times with 100% ethanol and air dried. The filters were one by one placed in 2 ml scintillation vials, and incorporated radioactivity was quantified using a scintillation counter.

### Data analysis

Methylation activity was measured as CPM by scintillation counting. The CPM was converted to Nm as previously described (53). Data were analyzed using the Prism software. For time-dependent kinetic measurements, data were fit to a linear regression of a nonlinear fit weighted by 1/Y2. A least square fitting method was used to plot the data for secondary plots, and the linear regression was not weighted. *K*_cat_ and *K*_*M*_ were determined by fitting the slopes from kinetic experiments to a nonlinear regression using the Michaelis–Menten formula in GraphPad Prism. The Z-factor comparing the performance of the filter and ELISA-plate assays were calculated as $Z=1-\frac{3\left(\sigma_{s}+\sigma_{c}\right)}{\left|\mu_{s}+\mu_{c}\right|}$, where $\sigma_{s}$ and $\sigma_{c}$ are the standard deviations of the methylation activity (CPM) with enzyme ($\sigma_{s})$ or without the enzyme ($\sigma_{c}$) (in the presence of the enzyme storage buffer with glycerol), and $\mu_{s}$ and $\mu_{c}$ represent the mean of the methylation activity (CPM) with and without the enzyme, respectively. The data analysis to determine IC_50_ values was performed by plotting percent of methylation activity at various AdoHcy (inhibitor) concentrations on the *x*-axis. Michaelis–Menten equation was used to fit data using variable slopes for enzyme inhibition. As described in the figure legends, errors were calculated as SE for three to six independent experiments. For $Z^{\prime }$, $\sigma_{s}\text{,}$ and $\sigma_{c}$ represent the standard deviation of methylation activity with and without the inhibitor AdoHcy.

## Data availability

All data are contained within the article, and raw data will be shared when requested at hgowher@purdue.edu.

## Supporting information

This article contains supporting information.

## Conflict of interest

The authors declare that they have no conflicts of interest with the contents of this article.
