# Supplementary material for: Development of a sensitive microplate assay for characterizing RNA methyltransferase activity: Implications for epitranscriptomics and drug development
Source: J Biol Chem. 2023 Sep 14;299(10):105257. doi: 10.1016/j.jbc.2023.105257 (PMC10582764; doi:10.1016/j.jbc.2023.105257)
Supplement: Supporting Figures S1–S4 [file mmc1.docx]

**SUPPLEMENTARY INFORMATION**

**Development of a Sensitive Microplate Assay to Characterize RNA Methyltransferase Activity: Implications for Epitranscriptomics and Drug Development**

Isaiah K. Mensah, Allison B. Norvil, Ming He, Emma Lendy, Nicole Hjortland, Hern Tan, Richard. T. Pomerantz, Andrew Mesecar and Humaira Gowher

**List of materials:**

Supplementary Figure S1-S4


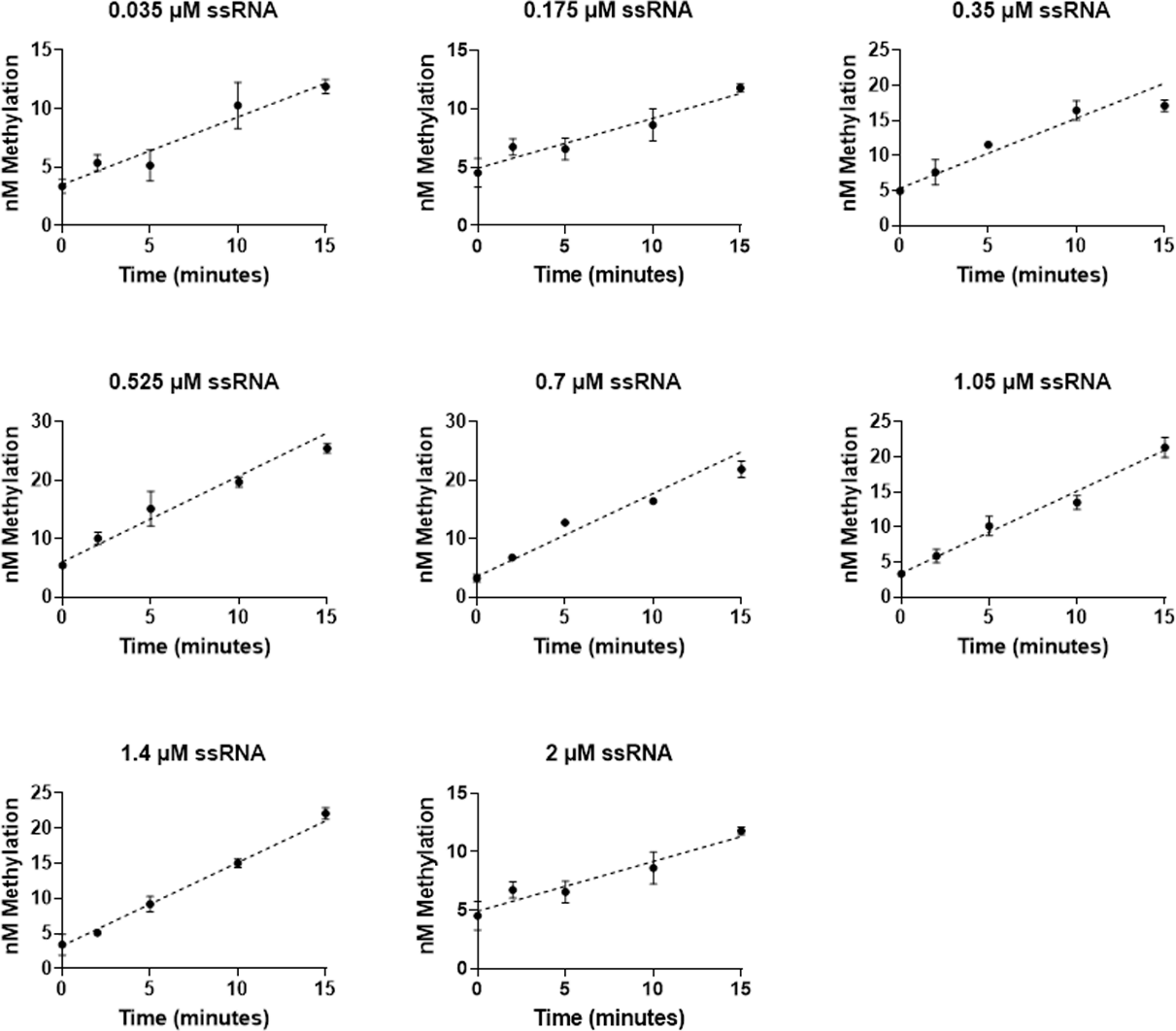


FIGURE S1**:** Enzyme kinetics of truncated ZIKV NS5 MTase using DEAE filter assay. Primary plots for the time course assays of RNA methylation. The assays were performed for 15 minutes, using varying substrate concentrations in the presence of 2 µM unlabeled AdoMet and 0.33 µM radio-labeled [3H] AdoMet. The reactions were initiated by the addition of the enzyme at 5 µM. The initial rate of reaction was determined using linear regression. The data shown are the average ± SD (n ≥ 2).


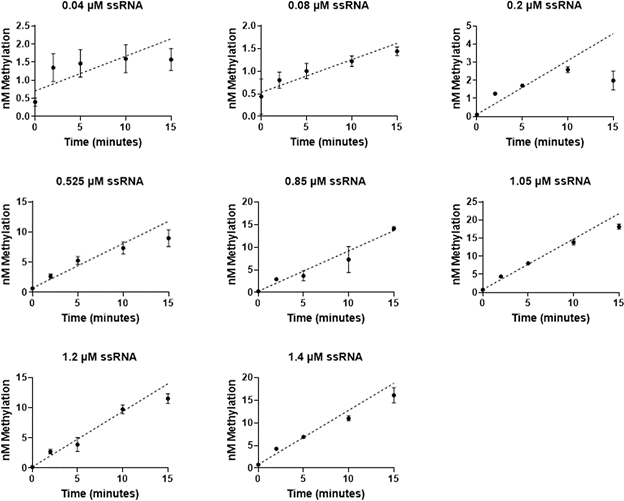


FIGURE S2**:** Enzyme kinetics of truncated ZIKV NS5 MTase using microplate assay. Primary plots for the time course assays of RNA methylation. The assays were performed for 15 minutes, using varying substrate concentrations in the presence of 2 µM unlabeled AdoMet and 0.33 µM radio-labeled [3H] AdoMet. The reactions were initiated by the addition of the enzyme at 5 µM. The initial rate of reaction was determined using linear regression. The data shown are the average ± SD (n ≥ 2).


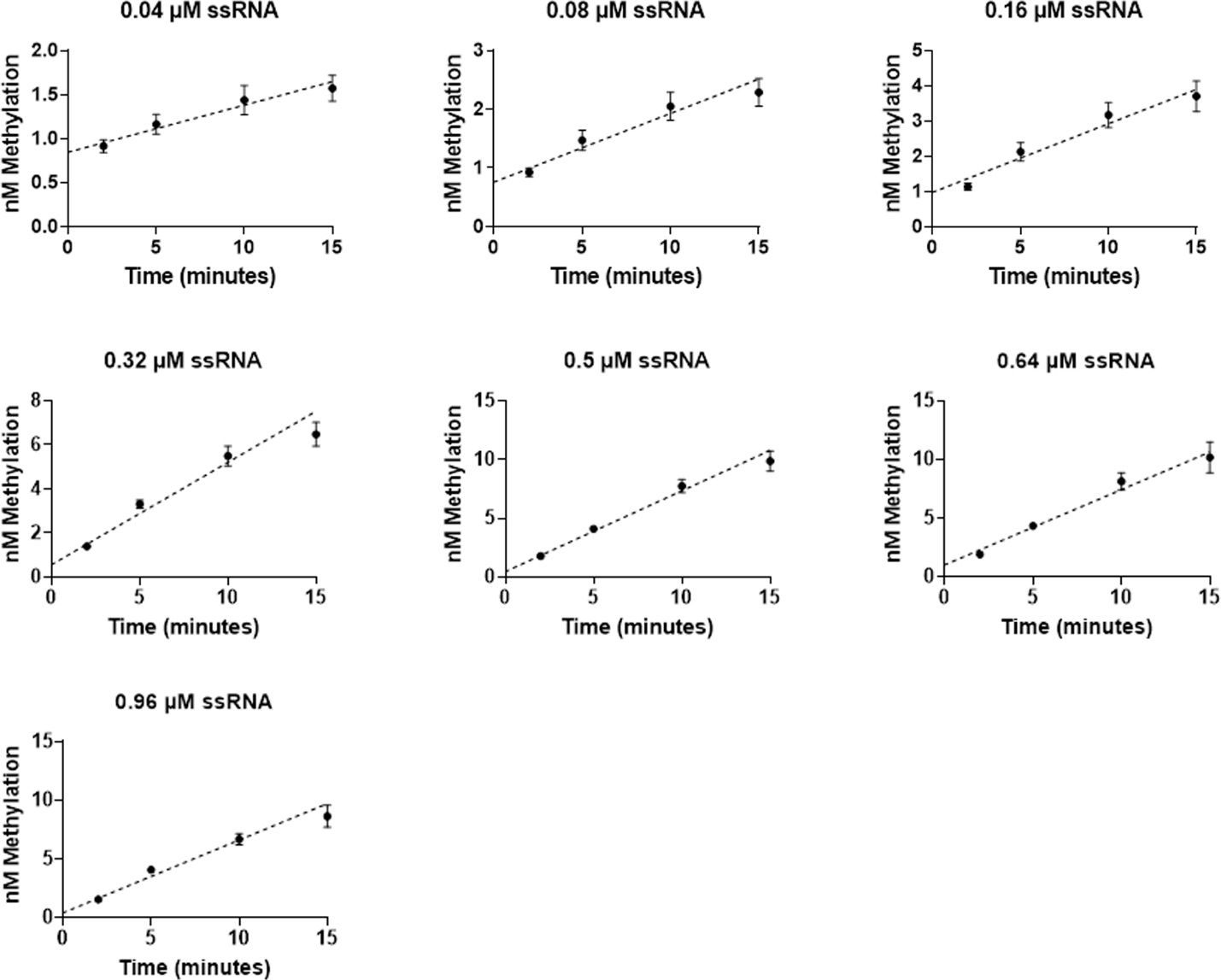


FIGURE S3**:** Enzyme kinetics of the full-length ZIKV NS5 MTase, using microplate assay. Primary plots for the time course assays of RNA methylation. The assays were performed for 15 minutes, using varying substrate concentrations in the presence of 2 µM unlabeled AdoMet and 0.33 µM radio-labeled [3H] AdoMet. The reactions were initiated by the addition of the enzyme at 5 µM. The initial rate of reaction was determined using linear regression. The data shown are the average ± SD (n ≥ 2).


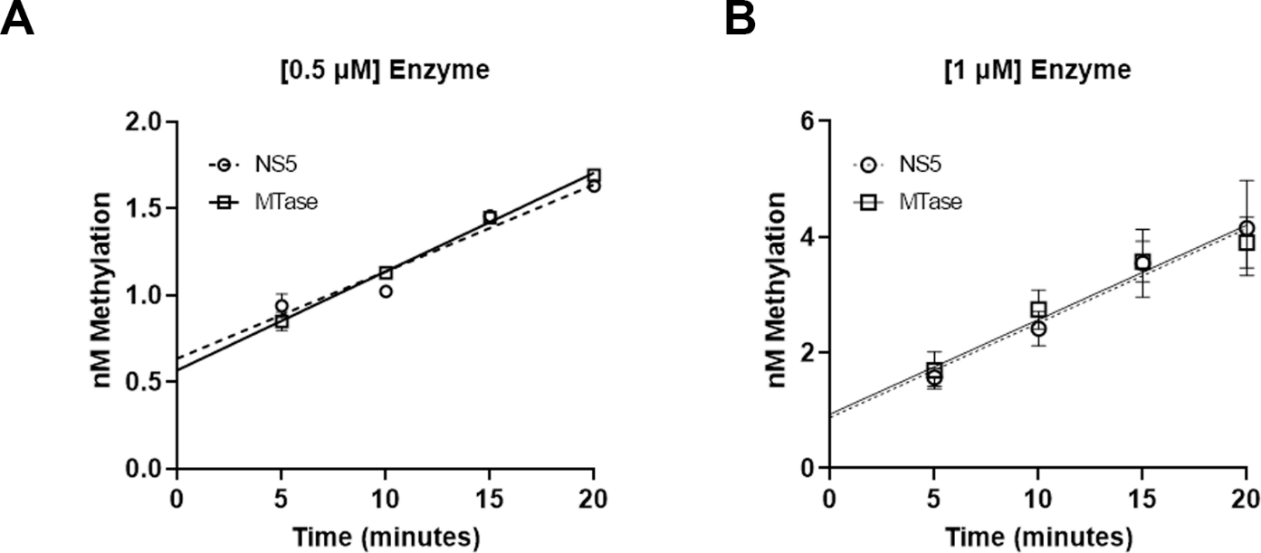


FIGURE S4**:** Steady-state kinetics of full-length and truncated ZIKV NS5. (A) Time course of methylation using full-length (NS5) and truncated (MTase) enzyme at 0.5 µM and 1 µM concentrations. The data fit a linear equation to measure the amplitude of the burst phase (y-intercept) and the apparent rate of methylated ssRNA release (slope). (B) Time course methylation assay using equivalent enzyme and substrate concentration at 1 µM. The data shown are the Mean ± SEM of n ≥ 3 independent experiments. Each independent experiment was performed in duplicate, and the average value for each data point was used to calculate the mean and SEM.
